# Fathers’ help seeking behavior and attitudes during their transition to parenthood

**DOI:** 10.1002/imhj.22008

**Published:** 2022-08-01

**Authors:** Amin Ghaleiha, Carrie Barber, Armon J. Tamatea, Amy Bird

**Affiliations:** ^1^ School of Psychology Faculty of Arts and Social Sciences The University of Waikato Hamilton New Zealand

**Keywords:** fathers and fatherhood, help seeking, mental health, transition to fatherhood, papás y paternidad, buscar ayuda, salud mental, Mots clés: Pères et paternité, chercher de l'aide, santé mentale, Väter und Vaterschaft, hilfesuchendes Verhalten, psychische Gesundheit, キーワード: 父親と父親であること、支援を求めること、メンタルヘルス, 关键词: 父亲与父亲身份, 寻求帮助, 心理健康, **الكلمات المفتاحية**: الآباء والأبوة ، طلب المساعدة ، الصحة النفسية

## Abstract

New fathers face multiple changes as they take on this complex, demanding, and continually shifting role. The current study aimed to understand these experiences, especially the ways fathers seek help and information while facing stressful situations. Eleven fathers completed a semi‐structured interview about their transition to fatherhood and whether and how they sought help and advice through that process. Results were analyzed using an inductive thematic analysis approach. Fathers viewed themselves as supportive figures and sources of financial and emotional stability for their families. Fathers experienced anxiety and uncertainty in their transition to parenthood, and utilized a variety of ways to cope with their stress; these were categorized into individual and interpersonal coping strategies. Most relied on their partner for emotional support, but some felt uncomfortable relying on her and using her for support while she was coping with pregnancy and new parenting. This study found that fathers tended to see themselves in a rather traditional role of provider and supporter of their partners and children, and this created some stresses for work‐life balance, and, for some, created a dilemma where they felt unable to seek emotional support from the person—their partner—on whom they would typically rely. These findings have important implications for fathers’ wellbeing and providing support programs for new fathers.

## Resumen

Papás novatos enfrentan múltiples retos a medida que asumen este papel complejo y exigente. Poco se sabe acerca de cómo los papás experimentan estos retos. Este estudio se propuso comprender estas experiencias, especialmente la manera como los papás buscan ayuda e información mientras que enfrentan situaciones estresantes. Once papás completaron una entrevista semiestructurada acerca de la transición a la paternidad y cómo buscaron ayuda a través de ese proceso. Los resultados se analizaron usando un análisis temático inductivo. Los papás se vieron a sí mismos como figuras de apoyo y fuentes de estabilidad económica y emocional para sus familias. Los papás experimentaron ansiedad en la transición a la paternidad y utilizaron diferentes maneras para arreglárselas con el estrés; estas maneras fueron categorizadas en estrategias individuales e interpersonales para arreglárselas. La mayoría confió en sus parejas para apoyo emocional, pero algunos se sintieron incómodos confiando en ella para ese apoyo mientras que ella se las arreglaba con el embarazo y la nueva crianza. En este estudio se encontró que los papás tendían a verse a sí mismos en un papel tradicional de proveer y de apoyar a sus familias, y esto produjo cierto estrés para equilibrar la vida laboral, así como un dilema en los casos en que se sintieron incapaces de buscar el apoyo emocional de sus parejas en quienes ellos confiaban típicamente. Estos resultados tienen implicaciones importantes para el bienestar de los papás y proveer programas de apoyo para los nuevos papás.

## Résumé

Les nouveaux pères font face à de nombreux défis quand ils embrassent ce rôle complexe et difficile. On sait peu de choses sur la façon dont les pères vivent ces défis. Cette étude s'est donné pour but de comprendre ces expériences, surtout les manières dont les pères cherchent de l'aide et des renseignements tout en faisant face à des situations stressantes. Onze pères ont rempli un entretien semi‐structuré sur leur transition la paternité et la manière dont ils ont cherché de l'aide au travers de ce processus. Les résultats ont été analysés en utilisant une analyse thématique inductive. Les pères se sont perçus comme des figures de soutien et des sources de stabilité financière et émotionnelle pour leur famille. Les pères ont fait l'expérience d'anxiété dans leur transition à la parentalité et ont utilisé différentes manières de faire face au stress; celles‐ci ont été catégorisées en stratégies d'adaptation individuelles et interpersonnelles. La plupart se sont appuyés sur leur partenaire pour le soutien émotionnel, mais certains d'entre eux n’étaient pas l'aise de compter sur elle pour du soutien alors qu'elle faisait face à la grossesse et au nouveau parentage. Cette étude a trouvé que les pères avaient tendance à se voir dans un rôle traditionnel de père pourvoyeur et de soutien pour leur famille et cela déclenchait des stress pour l’équilibre travail‐vie de faille, et un dilemme s'est avéré être qu'ils se sentaient incapables de chercher du soutien émotionnel auprès de leur partenaire sur qui ils compteraient typiquement. Ces résultats ont des implications importantes pour le bien‐être des pères et la création de programmes de soutien aux nouveaux pères.

## Zusammenfassung

Wie man zum Vater wird: Eine Exploration des Weges in die Vaterschaft neuseeländischer Männer

Junge Väter stehen vor zahlreichen Herausforderungen, wenn sie die komplexe und anspruchsvolle Vaterrolle übernehmen. Es ist wenig darüber bekannt, wie Väter diese Herausforderungen erleben. Diese Studie zielte darauf ab, diese Erfahrungen zu verstehen, und zwar insbesondere die Art und Weise, wie Väter in Stresssituationen nach Hilfe und Informationen suchen. Elf Väter absolvierten ein halbstrukturiertes Interview über ihren Rollenwechsel zur Vaterschaft und wie sie in diesem Prozess Hilfe suchten. Die Ergebnisse wurden mithilfe einer induktiven thematischen Analyse ausgewertet. Väter betrachteten sich als unterstützende Figur und Quelle finanzieller und emotionaler Stabilität für ihre Familie. Väter erlebten Angst beim Rollenwechsel zur Elternschaft und nutzten unterschiedliche Wege, um mit Stress umzugehen ‐ diese wurden in individuelle und zwischenmenschliche Bewältigungsstrategien eingeteilt. Die meisten verließen sich auf ihre Partnerin, um emotionale Unterstützung zu erhalten. Einige Väter fühlten sich allerdings unwohl dabei, sich auf die Unterstützung ihrer Partnerin zu verlassen, während diese mit der Schwangerschaft und ihrer neuen Elternrolle zurechtkommen musste. Die Studie ergab, dass Väter sich in der Regel in einer traditionellen Rolle des Versorgers und Unterstützers ihrer Familie sehen. Dies kann zu einer Belastung der Work‐Life‐Balance und dem Dilemma führen, dass sich Väter keine emotionale Unterstützung von ihrer Partnerin suchen, auf die sie sich sonst verlassen. Diese Ergebnisse haben wichtige Implikationen das väterliche Wohlbefinden und für Unterstützungsprogramme für junge Väter.

## 抄録

父親になること:ニュージーランドの男性の父性への道のりを探る

初めて父親になると、複雑で要求の多い役割を引き受けることになり、様々な課題に直面することになる。これらの課題を父親がどのように経験するかについては、ほとんど知られていない。本研究は、これらの経験、特にストレスの多い状況に直面しているとき父親が支援や情報を求める方法を理解することを目的とした。11人の父親が、父親になる移行とその過程を通じてどのように支援を求めたかについて、半構造化面接を受けた。結果は帰納的主題分析により解析された。父親は、自分自身を家族の支えとなる存在であり、経済的、情緒的安定の源であるとみなしていた。父親は、親になる移行期に不安を経験し、ストレスに対処するためにいろいろな方法を活用しており、これらの対処方法は個人的対処方法と対人的対処方法に分類された。ほとんどの人が精神情緒的な支えとしてパートナーを頼りにしていたが、相手が妊娠や初めての子育てに対処しようとしている中で頼るということに気まずさを感じるという人もいた。本研究で、父親たちは、家族を養い支えるという伝統的な役割の中に自分自身をみる傾向にあり、 そのことが、仕事と生活のバランス上にストレスを引き起こし、通常は頼れるパートナーに精神情緒的な支えを求められないというジレンマを生み出しているということが分かった。これらの知見は、父親の幸福や、初めて父親になる人への支援プログラムの提供に重要な示唆を与えるものである。

## 摘要

新任父亲在承担这一复杂而苛刻的角色时面临着多重挑战。关于父亲如何经历这些挑战, 我们知之甚少。本研究旨在了解这些经历, 尤其是父亲在面临压力时寻求帮助和信息的方式。11位父亲完成了一项半结构化访谈, 内容是关于他们向父亲身份的转变, 以及他们如何在这个过程中寻求帮助。结果采用归纳主题分析法进行分析。父亲们认为自己是家庭经济和情感稳定的支柱与来源。父亲在转变为父母的过程中会感到焦虑, 他们会使用不同的方式来应对压力;这些策略分为个人应对策略和人际应对策略。大多数父亲依赖伴侣的情感支持, 但也有些人在伴侣应对怀孕和养育新子女时, 会对依赖她的支持感到不舒服。这项研究发现, 父亲倾向于将自己视为家庭的提供者和支持者的传统角色, 这给工作和生活的平衡带来了一些压力, 并陷入了一种困境, 使他们感到无法从通常依赖的伴侣那里寻求情感支持。这些发现对父亲的健康以及为新任父亲提供支持计划具有重要意义。

## ملخص

الانتقال إلى الأبوة: استكشاف رحلة الرجال النيوزيلنديين نحو الأبوة

يواجه الآباء الجدد تحديات متعددة أثناء توليهم هذا الدور المعقد والمتطلب. لا يُعرف سوى القليل عن الكيفية التي يواجه بها الآباء هذه التحديات. هدفت هذه الدراسة إلى فهم هذه التجارب ، لا سيما الطرق التي يلتمس بها الآباء المساعدة والمعلومات أثناء مواجهة المواقف العصيبة. أكمل أحد عشر أبًا مقابلة شبه منظمة حول انتقالهم إلى الأبوة وكيف طلبوا المساعدة خلال هذه العملية. تم تحليل النتائج باستخدام التحليل الموضوعي الاستقرائي. اعتبر الآباء أنفسهم شخصيات داعمة ومصادر للاستقرار المالي والعاطفي لأسرهم. عانى الآباء من القلق أثناء انتقالهم إلى الأبوة واستخدموا طرقًا مختلفة للتعامل مع التوتر ؛ تم تصنيفها في استراتيجيات المواجهة الفردية والشخصية. اعتمد معظمهم على شريكهم للحصول على الدعم العاطفي ، لكن البعض شعر بعدم الارتياح للاعتماد على الشريكة للحصول على الدعم أثناء تأقلمها مع الحمل والرعاية الوالدية الجديدة. وجدت هذه الدراسة أن الآباء يميلون إلى رؤية أنفسهم يلعبون دورًا تقليديًا كمعيلين وداعمين لأسرهم ، وقد خلق هذا بعض الضغوط على التوازن بين العمل والحياة ، ومعضلة شعورهم بأنهم غير قادرين على طلب الدعم العاطفي من شريكهم الذي يعتمدون عليه عادة. هذه النتائج لها آثار مهمة على رفاهية الآباء وتقديم برامج الدعم للآباء الجدد.

## 1 BACKGROUND

The concept of fatherhood has changed for a new generation of fathers who are more involved in caring for their family and spending time with their children than their forefathers (Kaufman, 2013). For example, contemporary population research found that 73% of fathers believed they were more involved with their children than their own father had been with them. In addition, 58% wanted to be more involved in their child's life, and of these, 89% identified work‐related commitments as the main barrier to involvement (Morton et al., 2016). Work‐family conflict may be particularly evident in dual‐earner households, with men adopting more caregiving roles within families (Shafer & Wendt, 2015). Some have compared the modern male situation to when women entered the workforce in large numbers, while keeping their past roles such as being a mother (Aumann et al., 2011). Traditional gender roles portray men as the provider (Zuo & Tang, 2000), while modern norms of fatherhood include being caring and having a more hands‐on role in daily child rearing (Deave & Johnson et al., 2008). These two potentially conflicting factors (being the primary provider and modern norms of fatherhood) can put pressure on men, which may in turn negatively affect their mental well‐being (Kaufman, 2013). Fatherhood is a major life experience with possible positive and negative effects on men's mental wellbeing (Isacco et al., 2016). Positive impacts include shifts towards a healthier diet and reducing smoking and alcohol consumption and longer life expectancy for those having children after the age of 30(Shawe et al., 2019). With regards to possible negative outcomes, fatherhood has been associated with an increase in anxiety and stress, relationship conflicts and depression (Fletcher et al., 2006; Singley & Edwards, 2015). Meta‐analyses indicate that up to 10% of fathers are affected by depressive symptoms during pregnancy and in the first year after birth (Cameron et al., 2016; Paulson & Bazemore, 2010). A recent meta‐analysis indicated that marital and parental distress and maternal depression are important risk factors for fathers in the prenatal period (Chhabra et al., 2020). Poor health, stress, or lack of social and relationship support predict depression symptoms in the postnatal period among expectant fathers (Underwood et al., 2017). Men's well‐being has a direct impact on the well‐being of their parenting partners and their children (Bellamy et al., 2009). Therefore, it is important to understand paternal adaptation to parenthood and how to mitigate against these potentially negative outcomes for those at risk.

Given the conflict and distress experienced by many men in the transition to fatherhood, it is critical to understand how this might be ameliorated through help‐seeking. Many studies have examined help seeking but it still remains a complex construct without a commonly referenced definition or standardized form of measurement (Rickwood & Thomas, 2012). The World Health Organization (WHO) has defined help‐seeking as “any action or activity carried out by an individual who perceives herself/himself as needing personal, psychological, affective assistance or health or social services, with the purpose of meeting this need in a positive way. This includes seeking help from formal services – for example, medical staff, counsellors, or …programmes – as well as informal sources, which includes peer groups and friends, and family members” (Barker et al., 2007, p. 2). This definition is inclusive and relevant to the psychosocial situation of new fathers.

Existing literature highlights barriers for men in both formal and informal help‐seeking (Isacco et al., 2016), such as masculine ideology that promotes self‐reliance and conceals vulnerabilities (Addis & Mahalik, 2003) and a lack of father‐oriented interventions (Giallo et al., 2017; Robertson, 2007). This is reflected in fathers’ recruitment to parenting programs (Stahlschmidt et al., 2013). Not only are fathers faced with barriers with regards to utilizing and accessing these programs, but they also have low turnout to programs that they enroll in (Fletcher et al., 2006; Weinman et al., 2005). Most mental health interventions for new fathers have been designed for partners of women with perinatal depression (Davey et al., 2006; Hynd et al., 2005; Kowalski & Roberts, 2000).

There is some evidence that new and expectant fathers have been concerned about the scarcity of structured support for fathers and the lack of father specific programs with the aim of answering their own needs (Davey et al., 2006; Friedewald et al., 2005). A study by Deave and Johnson (2008) found that first time fathers typically felt ignored and frustrated at being excluded during the antenatal and postnatal period. Other barriers to fathers’ help‐seeking include a clinic's atmosphere and attitude; for example, some male clients could perceive the female dominated environment of social services as uninviting (Lee et al., 2011). The maternity and infant care workforce is typically majority female, so that there are very few male professionals that fathers will encounter in their interactions as a new father. The absence of male clinicians and mental health workers may also discourage men from seeking help, since some men prefer other men for counselling (Shafer & Wendt, 2015). Despite a growth in researching fathers’ mental health and needs, it remains an understudied field (Giallo et al., 2017; Isacco et al., 2016). In particular, there is relatively little research available on fathers’ help‐seeking behavior and attitudes during the transition to fatherhood.

While research and clinical work on perinatal mental health has traditionally focused on mothers (Åsenhed et al., 2014; Fletcher et al., 2006; Weinman et al., 2005), some researchers have highlighted the perinatal period as an important opportunity for health care providers to engage with fathers (Panter‐Brick et al., 2014). Fathers who participate in antenatal visits and classes may be open to examining health behaviors, and perhaps increasing awareness of the health and social services available to them. The aim of the current study was to examine the transition to fatherhood, positive and negative impacts, how fathers cope and seek support with these changes, and where they get information, support, and advice. The focus was on understanding barriers and potential opportunities to help‐seeking for fathers, specifically the transition to fatherhood as a particularly challenging time. We were interested in finding out what fathers need help with, including but not limited to psychological distress, child rearing and relationships.

## 2 METHODS

### 2.1 Recruitment

Participants had to be at least 18 years old and live in Waikato region of New Zealand. Eleven participants were recruited during 2018 and 2019 through noticeboards, social media, word of mouth, and paper advertisement distributed at the local university, daycare centers and other community centers such as sport clubs.

### 2.2 Sample characteristics

All the participants were male fathers with female partners and born in New Zealand. Seven participants identified as Pākehā (New Zealand Europeans) and four as Māori (indigenous people of New Zealand). They ranged from early twenties to early forties in age (*M*_age_ = 33). Seven participants had university degrees. Nine were employed and two were full time university students, and most (9 out of 11) lived with their partner. The participants had one or two children aged 13 or below. The average age of the participants’ children at the time of interview was 4 years old and their age ranged from 6 months to 13 years old.

### 2.3 Procedure

This study was approved and overseen by Human Research Ethics Committee. A semi‐structured interview guide with open‐ended questions was used to explore participant experiences as fathers. The interview followed a chronological order (from conception through the first few weeks postnatal and beyond the immediate neonatal period). Participants were asked about their involvement in caring for the baby, their interaction with maternity care providers, change in the family dynamic after the birth of their child, coping with stresses of parenthood, what they liked about being a father, and the ways they got information and advice. The questions were adapted in response to information provided during the interview. The interviews were audio‐recorded and transcribed verbatim. Participants were given the opportunity to check and correct or amend transcripts. Interviews were conducted face to face by the first author in locations of the participants’ choice. The beginning of each interview was dedicated to introducing the topic and building rapport with participants. They were assured that all the provided information would be regarded as highly confidential, and no identifying information would be included in any dissemination of data; pseudonyms were assigned to participants and used in all reports. Fathers were quite open about sharing their experiences.

### 2.4 Data analysis

Thematic analysis was used to understand the transcribed interviews. Qualitative analytic methods are appropriate for understudied topics and exploring relationships between themes (Bailey, 2007; Patton et al., 2002). Thematic analysis also provides a great degree of flexibility which allows for themes to be formed in a variety of ways, and further helps in interpretation of those themes supported by data (Braun & Clarke, 2017). Braun and Clarke's (2017) six steps were used; transcripts were read and inspected, preliminary codes were generated and assigned to the data, themes and patterns were identified across the transcripts, themes were reviewed and discussed with co‐authors, each theme was named, and finally, themes were described, presented, and analyzed. Coding began after four interviews were conducted, and data collection was halted when saturation was achieved. The NVivo software program (Woolf, 2017), a qualitative data analysis tool for analyzing rich‐text and data, was used to code and categorize the data into key subthemes and themes. The coding was conducted by the first author and the results were shared with the second author who provided feedback on the initial codes. Repetition of information in fathers’ accounts and the codes was observed by the time the 10^th^ and 11^th^ participant were interviewed. An inductive approach was used in which common and important patterns were identified. In this approach, the emerged themes are strongly linked to the data and are not driven by a predetermined theoretical framework (Clarke & Braun, 2017).

## 3 RESULTS

Results are presented under two main themes (see Table 1) centered on the key issues identified by fathers: (1) Being a support for my partner; and (2) managing stress. Each of these themes and the four subthemes within them emerged from answers to research questions regarding perceptions of men about their role as a father and their experiences in the context of fatherhood and parental help‐seeking. Relevant quotes have been selected to illustrate the main findings.

**TABLE 1:** An overview of the themes

Themes	Subthemes	Characteristics
Being a support for my partner	I ca not do both	The conflict between being a provider and being a supportive father/husband
	I am a father: a new role	Fatherhood a significant change in life, an identity, and a source of pride and joy
Managing stress	Finding someone to talk to	Sharing their struggles with another individual or seeking help from someone
	You just got to deal with it	Fathers’ ways of coping with stress that did not involve any other person. This reflected suppression of emotions, as well as helpful (e.g., exercise) and unhelpful (e.g., drugs or alcohol) intrapersonal ways of coping.

### 3.1 Being a support for my partner

Most fathers described their role as being the supportive figure for their partner. They viewed their partner and their baby as the center of attention. Their role was defined by providing financial, psychological and emotional support for their partners and, later, their family.

Matthew discussed the importance of providing emotional support and reassurance for his wife during distressing times for her (pregnancy and especially birth).

yeah, like there's a lot of emotional support necessary and reassurance and I think it's kind of the main thing is like when hormones are running rampant, being a figure of stability

Fathers sometimes felt ineffective in providing this emotional support for their partners. The birth experience was described as quite overwhelming; they knew they were supposed to comfort and support their partner, but they were not sure how.

Lucas: I didn't know what the heck I was supposed to do so I just stood there, getting told what to do and getting told off, [sighs] it was ridiculous. It was stressful and as I mentioned it was a little bit unnerving, didn't really hundred percent know how to help my wife who was going through so much pain. I felt useless because I couldn't help her in any way as she was going through all of it…

Some midwives and other healthcare providers also seemed to perceive fathers’ role as being the supportive figure for mothers, and catering to mother's needs. Fathers were reminded that they must keep a close eye on their partners and offer as much help as they could. When asked about what subjects were discussed with the midwife, Aaron said:

Yeah, just mother and baby really and also how I could support so making that sort of say to me “make sure she's eating, make sure you're keeping her hydrated” and just make sure you help out, so the emphasis towards me was… I suppose keeping me in line with helping, yeah that was the main goal, I would say.

When asked about their interactions with midwives, most participants did not express concern about the fact that midwives or other healthcare professionals did not ask about their wellbeing and focused solely on the mother. They expressed the belief that the mother and the child were the priority and the focus should be on them, putting their own needs aside as part of the support role

Oliver: I didn't really exist heavily but I was fine with that at the same time because you know it's about the baby… she [the midwife] was a bit more clinical in her approach but you trusted her…

#### 3.1.1 I cannot do both

This subtheme captures the conflict between being a provider and being a supportive father and partner. Most participants expressed an interest in being involved in supporting their partner and newborn child; however, work commitments were seen as a major barrier. Most fathers tried their best to go to scans, antenatal classes, and hospital appointments, but felt pressure and conflict between the demands of their work and need to be the breadwinner and the needs of their partner and child.

The birth process, difficulty with feeding, and sleep deprivation were significant sources of stress and exhaustion, that were experienced alongside on‐going work obligations.

Oliver: She got an epidural and that was after the first 24 hours because she needed to sleep. She didn't really want to but she ended up doing that and so when she was sleeping I was studying, trying to get ready for the exams but… yeah so the birth process was quite stressful.

Sean stated that he was under a lot of pressure due to the nature of his profession and found it difficult to juggle both work and parental responsibilities. Nonetheless, he was mindful of his partner's stress and the struggle of taking care of the baby at home all day.

As soon as I come home my partner is shattered, she wants the baby off her and I've literally just finished work. I have just been working in [workplace] all day, I kind of just want to sit down for five minutes but I kind of forget that she wants to sit down for five minutes as well…

### 3.2 I am a father: A new role

Being a father was also a shift in identity for most participants. They expressed love, pride and joy in being a father. Fatherhood was also a significant change in their lives; they spoke of putting their children before themselves and prioritizing their needs.

Mateo has been a parent for more than 10 years. He felt proud to be a father and hearing his extended family commending him for being a great father and praising his daughters was important to him. He added that hearing those comments meant a lot because he knew he had contributed to their upbringings. He explained that he was faced with a choice, either to be absent and only care about his own enjoyment or to “step up” as a father. He chose the latter.

Then hearing family members and whānau [family and extended family] saying your girls are so awesome. To a father that makes you feel proud because you have had a hand in bringing those kids up… It was an unplanned baby… but when I thought wow – I've been given a lifeline here, I've been given a gift, a taonga, pounamu [a treasure, traditional carved stone]– use it. What would you rather do? Go inside for the rest of your life or drink and be idiots or step up and be a father. So I stepped up and be a father. Even my father saying proud of you son, the dad you have become and you haven't followed my footsteps and you have done your own path.

Some fathers agreed that most of the attention of healthcare professionals should be dedicated to the mother and the baby; however, they felt pushed aside and felt that the father‐child relationship was being ignored.

… For the most part I was pretty happy with the experience and I think in New Zealand we are particularly lucky… like there could be an argument to be made that there is no focus about bonding, and dads are very much pushed to the side. It's about mums and it's about the birthing process and it's about the children……

In summary, fathers saw their role as being supportive, and it was their duty to provide for their family. They also wanted to spend more time with their children, and help with childcare, but struggled with being both a provider and a "good dad." These interviews revealed that most men were comfortable with the main focus of midwives and healthcare professionals should be on the mother and the baby; however, this left some fathers feeling uncertain about how they could help, and not knowing where they could get support for themselves.

### 3.3 Managing stress

This theme explores some of the difficulties with parenting, and how men countered these stressors. Fatherhood brought both joys and difficulties. Men viewed family, friends and healthcare providers as major sources of support as they became parents.

Both parents had to adapt to a new a lifestyle and routine. Most fathers reported that the first few weeks after birth were quite difficult.

Benjamin: The hardest thing I've ever done in my life. The first three days we didn't really sleep. My wife didn't sleep the night the baby was born or the next day. She went about 2.5‐3 days without sleep. For me, I maybe got a half hour here and there, but every time we put the baby down to sleep and then she started screaming again. I would compare it to some kind of psychological torture…

Cooperation and teamwork meant some fathers could rest, regain their energy and continue supporting their partner and child.

Benjamin: So I was very fortunate, my wife and I work well as a team, so if I'd had enough I could just give the baby to her and if she'd had enough she could give it to me, and we would work as a team.

Being a father was also a source of enjoyment and pride for fathers. Many participants talked about their love for their new baby and despite all the hardship, they loved their family and were glad to be a father. Recognizing this love and connection was described as ameliorating the stress.

Benjamin: I guess, yeah, you get the stress and the anxiety and the pressure and everything builds up, but then you have those moments of looking at this precious little angel and I don't know, it all just melts away.

Although pregnancy can unite a couple, it can also lead to conflict. Aaron talked about struggling with depression, not feeling connected to his spouse after his child's birth, and seeking formal support.

I suppose we weren't connected to each other and connected as a family…

I: As a result of you being away?

Being away and probably not being present when I was at home and I sort of found out now it was probably because I was struggling with depression and anxiety and I went into a big meltdown I guess so from there I went to see just a counsellor.

Fathers experienced several stressors during their transition to parenthood, such as work‐life balance, financial issues, and concerns about the health of their partner and baby. They described different ways to deal with their stress. These could be grouped into two broad subthemes: interpersonal, help‐seeking strategies; and individual (or intrapersonal) coping strategies.

#### 3.3.1 Finding someone to talk to

This sub‐theme describes the ways in which fathers shared their struggles with another individual or sought help from someone.

Partners were the main source of support for men. Some participants found it easier to talk to their partners than to other people, both during and after the pregnancy. They trusted their partner more than anyone else, and they also relied on their knowledge for anything child care related.

Jack: Because obviously I trust her… she does her research, she is very clued up on everything like that. She has always got an answer if I have a question really. So… yeah, I honestly don't look outside of her most of the time.

Isaac: If I had something to get off my chest I'd still talk to Sarah.

Sean: It kind of gets to a point where I crack once in a while, and finally talk to her and my partner says look, you know you can come talk to me and I say to her, you are stressed out [after the pregnancy] as enough as it is…

Matthew preferred to talk to his friends rather than his partner and family (parents) about his problems, because he did not want to worry his family.

I would probably divulge more to friends than I would to my family. Just because you know, like I want for them to feel like it's going okay…

Aaron sought support from professional mental health services. He had suicidal thoughts after an argument with his partner. He sought help from his general practitioner due to severity of his distress.

We had a really rough night the night before and then I started having like suicidal thoughts on that day…and then that Monday I decided I had to go to the doctor to change some things in my way of being. They put me on antidepressants, they got psychologist organised…

Family and friends provided a range of practical, and emotional support. Mothers and mother‐in‐ laws played an important role in providing support for the partner, relying on their experiences as being mothers themselves.

Mateo: The mother‐in‐law and my sister‐in‐law and my mum and whānau [family] were always there. The mother‐in‐law was always on the phone if we needed anything.

Isaac: We had some friends of ours move in and so it was like any kind of support that we needed, people checking on me… They were able to just jump in and take care of the kids or to be involved in what we were doing and we really felt supported.

Oliver: My mum being a nurse, you know we trusted her information so she was really heavily involved. She is really child orientated obviously so we got a lot of good information from [her]

Effective support from healthcare professionals reduced distress for fathers and their spouses during and after pregnancy. They were also an important source of knowledge for fathers.

Lucas: My parents they were telling me how to be supportive, just how to support but the midwife was really the one that taught me about the process and what was happening and how it's going to go, how everything is going to happen.

Telephone health information helplines were recommended by a number of participants. They explained that knowing there was someone who provided relevant information and reassurances was a huge relief.

Bill: what has been amazing was the Plunket [Child health agency] Helpline.

Yeah, so we rung them when we didn't know what was happening or sick or something, we would ring them a lot and they were very helpful

Negative experiences with healthcare providers exacerbated stress in fathers. Sean was suspicious of their midwife's practice, implying that she may have not recommended hospital to secure her monetary gains.

We heard later on that apparently if… they have to induce or there is any hospital intervention the midwives actually get paid less. So we're not sure if that has anything to do with it

He also noted that there was no psychological support offered by healthcare professionals for the fact that his partner was struggling to feed the baby due to a medical condition. Sean explained that it took a heavy toll on his partner.

We had issues with feeding… he had the tongue tie… Her nipples were really really sore because he couldn't latch properly and he was just damaging them to hell. So we got a lot of support with that but… there was no mental coping strategies [for her] that were introduced about it. Like there was a lot of “ah here is nipple shields… keep trying”, there was no… That's the thing especially my partner, it really got to her mentally

In summary, some fathers relied on a variety of people, including their partners and friends to seek support. The support they sought from the health professionals was mostly in relation to their baby's or partner's health, rather than their own wellbeing.

#### 3.3.2 You just got to deal with it

This subtheme refers to fathers’ ways of coping with stress that did not involve other people. Some, such as Oliver, described using drugs or alcohol to manage their stress:

Oliver: At the time you just got to deal with it… I'd just have a cone [cannabis] before I went to bed. I don't anymore and I have cleaned up but just during it I felt that I needed something

Some, like Mateo, exercised to reduce their stress.

I would sort of every now and again probably just go to the gym. I'm a pretty active person, so the gym was probably my solitude of trying to let some stress out if there was no one around.

Other participants framed some of the stressful situations they experienced as "normal" at the time. They gave little weight to their distress and claimed that they were not "severe" enough to require immediate or professional help. They normalized the difficulties they experienced; for example, Benjamin mentioned that everyone deals with these stressors, so why would his situation be any different. He also referred to a Kiwi saying “she'll be right.”

I never went to a GP about any of that. I don't know, it just goes back to that you just assume it is normal. Everyone has got to deal with it, so why should your case be any different? Maybe it is that New Zealand mentality of she'll be right, harden up.

Most fathers did not feel that their stress or distress was unusual or indicated a need for professional intervention. Some were more comfortable with seeking informal help (e.g., talking to family and friends) but even that posed a problem; those who sought help from family were worried about talking behind their partner's back. Some felt that sharing their relationship problems with their own family would alter the family's perception of their partner and would create animosity between them. Therefore, most participants did not share many of their specific concerns or distress with others, even when they had approached them for support.

*Sean: there is a lot of personal stuff that she didn't want my mum knowing really but I needed to talk about it [but I did not] …*

George: No just each other mostly. Occasionally parents, but wouldn't be like, like she could go talk to her mum completely in depth and that's fine, whereas me with my parents, not so much, like I can still talk to them, but, I be careful what I say because I don't want to alter their opinions on anything…

Partners were the main source of emotional support, but fathers felt guilty expressing their own needs when their partner was heavily pregnant or had just given birth. Some participants felt it was "wrong" or "selfish" to seek emotional support or affection from their partner.

and she still says come talk but I still can't bring myself to put my feelings on her… especially when sometimes my needs do feel a bit selfish, whether not getting any affection from her or cuddles because she has got the baby on her, little things like that

There are a variety of different approaches to managing the stress of new fatherhood. Families offer help and support, but they can be experienced as intrusive, and some men were protective of the impressions they might develop if they talked frankly. Some fathers appeared to be cautious about seeking help because of concern it might cause conflict or threaten their privacy. Some fathers were happy to rely on healthcare professionals, while others did not feel their problems merited this step. For many men, the partner was the primary source of support, but also one who they saw as stressed, and so they were hesitant to burden her, and turned to more individual coping strategies.

## 4 DISCUSSION

The aim of this study was to explore the transition to fatherhood, positive and negative impacts, how fathers cope and seek support with these changes, and where they get information, support, and advice. The study was based on the view that fatherhood is an important transitional period for men, and little in‐depth qualitative research has previously been dedicated to well‐being of new and expectant fathers compared to mothers (Wong et al., 2016). We found that fatherhood is associated with many positive and negative experiences, such as bonding with the baby, and feeling distressed by demands of parenting. A novel finding was that some fathers described having limited ways of seeking help, and their mainstay—their partner—was not perceived as available at this crucial time. Fathers also experienced difficulty with balancing their role as providers and fulfilling their responsibilities as modern fathers.

### 4.1 Stress of two roles

Being a provider and trying to be a caring father at the same time was identified as a source of stress for some fathers. It was very important to men in our study that their family was financially secure. Most men in this study perceived themselves as the support figure for their partner and child. They provided different types of support but identified their primary roles as being the breadwinner and a source of emotional stability. Fathers reported that others, such as midwives, their in‐laws, and their own families, also viewed this as their primary role. These findings suggest that fathers are retaining their traditional role as breadwinners, as well as adopting additional responsibilities (the care of children in particular) (Churchill & Craig, 2021; Williams et al., 2013). These new roles and responsibilities during the transition complicated their personal journey to fatherhood.

Childbirth was described by fathers as a completely new experience. Swedish fathers have found their presence in the birthing room more demanding than originally anticipated; they particularly felt unprepared for the delivery process, their own reactions, and their partner's agony and pain (Ledenfors & Berterö, 2016). Similarly, fathers in this study described feeling out of their depth and uncertain of how to help during the process of labor and delivery. Many fathers in our study also found the first weeks after birth very tiring and stressful, due to sleep deprivation, learning new parenting skills, and adjusting to a new lifestyle. Fathers emphasized the importance of cooperation with their partners when it came to childcare. These findings appear to reflect the changing role of modern fathers (Aumann et al., 2011; Deave & Johnson, 2008). Men in this study described themselves as supporting their partner in childcare. Their partners would reciprocate, so men could rest before going to work. This was an effective way for fathers to bond with their family, fulfil their role as the support figure and to receive support from their partner to reduce their fatigue.

A study of Israeli divorced custodial fathers showed that despite facing similar tasks and obstacles as their parenting partners, fathers sought and received less help (both formal and informal) during and after divorcing their spouse (Cohen & Savaya et al., 2000). The authors attributed this phenomenon to the importance of self‐reliance and independence to fathers. Similarly, the new fathers in our study often hesitated to reach out for help—“You've got to deal with it.” Many viewed fatherhood as an identity which involved putting their partner and family's needs above their own (e.g., working long hours and sleep deprivation). For participants, making these sacrifices was an admirable act and built resilience. This conceptualization made coping with anxiety and sleep deprivation easier. Being a father gave participants a sense of purpose. This finding is in line with the positive psychology/positive masculinity (PPPM) model (Englar‐Carlson & Kiselica et al., 2013); this strengths‐based approach aims to help male clients identify positive aspects of their masculinity (e.g., worker‐provider, male ways of caring, or self‐reliance) and thus use them to improve their wellbeing (Englar‐Carlson & Kiselica, 2013). Fathers may respond well to conversations centered on the importance of self‐care to be able to fulfil their fatherly roles. Health professionals could use these conversations to encourage fathers to seek help by describing appropriate help‐seeking and self‐care as a responsibility, rather than a deficit, for new fathers.

Many participants reported working hard, and trying to give their partner a break, accompany her to appointments and scans, and be involved in infant care (e.g., putting the baby to asleep). Our findings concur with existing research highlighting work‐family conflict as a primary concern for contemporary fathers (e.g., Shafer & Wendt, 2015). Work‐family conflict may continue to increase as fathers adopt more childcare responsibilities while maintaining their role as the financial provider (Allard et al., 2011). Father‐oriented interventions can address barriers (e.g., women‐centered prenatal support, lack of fathers’ participation in health services) (Giallo et al., 2017; Robertson, 2007) by identifying and accommodating fathers’ needs. This could include more flexible parental leave for fathers and educating employers about the stress of fatherhood. Antenatal classes and healthcare providers can also focus on aspects of wellbeing that are important to fathers such as the ability to provide care and support for their family (Darwin et al., 2017). Furthermore, healthcare providers could conduct wellbeing checks for fathers and lead conversations around paternal wellbeing and acknowledgment of the difficulties of being a new parent. Future research could explore the types of help and the modes of help preferred by fathers, as well as beliefs and attitudes on masculinity and their relation to potential barriers to help‐seeking. The impact of these help‐seeking and other coping behaviors on mental health across time could also be examined using a longitudinal quantitative design.

### 4.2 Dilemma of closeness

Men sometimes find it difficult to know when and where they can seek help regarding their day‐to‐day distress. There is evidence that men's intimate partners are often their main source of emotional support (Åsenhed et al., 2014); however, mothers may not be able to fulfil this role during pregnancy and the postnatal period, as both parents are focusing on caring for their new‐born. This was consistent with the experiences described by fathers in our study; for most, their partner was their main source of emotional support, and many felt unable to lean on her during this challenging time. On the one hand, this speaks to the closeness of these relationships; however, when fathers rely exclusively on their partners, they risk being unsupported in one of the most stressful transitions of life.

Our findings show that friends and extended family also played an important role in supporting fathers; some of their support was practical (e.g., helping with childcare just after birth), and some was emotional (some fathers shared their problems with family and friends). However, there were limitations on these sources of help: fathers described their friends’ inability to relate because of their lack of shared experience, and they worried about fomenting criticism of their family toward their partner. This presents a dilemma for these men, whose most usual sources of interpersonal support are not seen as available at this key time. Social support has a significant impact on fathers’ mental health; fathers with effective social networks are less likely to experience parenting and marital conflicts (Aycan & Eskin, 2005). Limited social and emotional support can add more stress to a father's life, which is associated with an increase in psychological disorders (Paquette, 2004). Considering fathers’ overwhelming reliance on their partners for emotional support, mothers’ experiences of postnatal depression may, in turn, increase fathers’ vulnerability to psychological distress.

In absence of usual interpersonal support, fathers used other methods to cope with stress. For example, substances (marijuana and alcohol) were used as the primary coping mechanism by some of the participants. Recent studies which have examined men's coping mechanisms with regards to psychological problems have found that substance use and other risky behaviors seem to be correlated with psychological suffering (Bilsker et al., 2018; Liu & Iwamoto, 2007). These coping behaviors have been attributed to masculine ideologies that endorse masking or ignoring negative feelings and avoiding reliance on health professionals and other people (Hammer et al., 2013). Such beliefs, if combined with isolation and absence of meaningful relationships, can increase the risk of suicidal behavior (Bilsker et al., 2018). The current findings extend extant literature and provide a window into potential reasons behind these coping strategies. Many fathers normalized and gave little weight to their stress and problems. The idiom “she'll be right” was used; it is a popular idiom in New Zealand which means most problems will right themselves with time. The participants were quite descriptive about their distressing experiences, and their impacts on their wellbeing, but most did not view their situation as "severe" enough to seek formal or informal help. This phenomenon may have occurred because fathers were simply prepared for the stress and may not have needed help (Palkovitz & Fagan, 2021) or perhaps they were not aware of the ways that stress can cause problems in health and wellbeing, and did not know what levels and types of stress are problematic and can be improved by seeking help and learning new strategies to cope.

Health professionals were an important source of knowledge for fathers. Many participants recommended Plunket Line (a free parent helpline in New Zealand which provides information on children's wellbeing and health). They also sought advice from midwives; however, most of this help‐seeking was focused on the health and welfare of the infant or partner, rather than their own wellbeing. Providing information about how health and social services, as well as informal (such as peer) support can provide both practical and social benefits might encourage fathers to expand their help‐seeking repertoires.

Midwives play an important role in prenatal care and childbirth in New Zealand; participants who had a positive relationship with midwives described a more positive transition to fatherhood. Men respected knowledge and seemed more likely to form a positive relationship with the midwife if they perceived her as competent. Ledenfors and Berterö (2016) looked at first time fathers’ experiences of normal childbirth, and suggested that good communication with the midwife reduced fathers’ fears and vulnerability. For our participants, birth was a new and potentially stressful experience. Fathers’ expectations from pregnancy and birth might not match the reality after the child's birth. This mismatched expectation is considered a risk factor for paternal depression (Chhabra et al., 2020). Maternity caregivers could help by providing information about realistic expectations and strategies for coping that address the needs and roles that new fathers are growing into.

Our fathers reported that fathers’ well‐being or mental health was not discussed by most healthcare professionals. They understood the reasoning behind the emphasis on mother and infant's health; however, some felt their needs as fathers were not recognized. Most available resources on pregnancy and childbirth are mother‐oriented, and fathers are often depicted as "outsiders" (Wong et al., 2016). Engagement with pregnancy‐related health services may be a missed opportunity to engage fathers. By establishing a rapport with fathers, maternity caregivers can help create a positive birth experience for both parents, which in turn may increase fathers’ child care involvement. Healthcare and maternal health services could initiate conversations with both parents about the stresses of being a new parent and share resources about parental mental health. Antenatal classes and postnatal depression support groups could organize additional support groups for new fathers. Men are more likely to adopt norms that are modelled by other men (Carli et al., 2001); therefore, parental services can utilize peer support to better engage fathers. These findings add to the current literature and highlight the importance of preparing and educating fathers for birth.

This study indicated how fathers’ perception of mothers’ "unavailability" during pregnancy and the postnatal period discouraged them from seeking emotional support from their partners. Future studies could explore the accuracy of this perception (e.g., fathers may be overestimating their partners’ distress; mothers might welcome fathers to share their struggles with them), and perhaps investigate how to guide fathers through what may seem a daunting task of sharing their own concerns with an exhausted, perhaps prickly partner.

Future studies could also explore when fathers think it is appropriate to seek support and what factors affect their decision to do so. There is a lack of research on stigmatization of help‐seeking but the available studies show that it is more common among men than women (M. E. Addis & Mahalik, 2003). This kind of stigma has real and major consequences for help‐seeking acts. As a result, men may mask their distress and attempt to control how they are perceived by others (M. Addis, 2011), missing out on help at a critical time in the family life cycle.

### 4.3 Limitations

This study included a small group of fathers who volunteered to discuss their experiences; therefore, the findings cannot be generalized to a broader population, but provide some in‐depth insight into the experiences of these men. For some fathers, this was up to 13 years ago and their recall may have been impacted by retrospective bias. These participants volunteered for a study knowing they would be talking about the experiences of early fatherhood. It is possible that fathers who are less reflective or less focused on their identity as fathers were less likely to volunteer for the study. How fathers coped at the time of their transition to parenthood (stress and conflict over their new role) could influence how they later recall their experiences. Events that have occurred since this transition, such as marital separation, child development, and the trajectory of fathers’ own mental health could all influence fathers’ retrospective recall.

Lastly, there is a notable gap in research on the experiences of men from different socioeconomic and cultural upbringings. The participants tended to be more highly educated than the general public, but did include four who identified as members of New Zealand's Indigenous Māori culture. Our findings do not inform us about more gender‐diverse parenting situations. While this study focuses on male fathers in heterosexual relationships, same‐sex parenting partnerships and/or non‐binary parents may also adopt a father role. Further research with these groups will help to tailor supports within perinatal care for fathers.

## 5 CONCLUSION

Fathers described themselves as financial providers for their family, and a figure of stability and emotional support for their partner, while also navigating their own personal journey to parenthood. Their roles and responsibilities were reinforced by partner, community and professional expectations. Fulfilling these responsibilities was challenging for them, but they relied primarily on internal and informal supports. Our participants used a variety of ways to cope with their stress; most were individual and some interpersonal. Fathers tended to downplay and normalize their distress, despite describing intense pressures and strong feelings.

Fathers’ primary source of support was almost universally their partner, and this presented a dilemma, as they felt it was not appropriate for them to rely on their partner when she was also experiencing stress. For some, this meant reliance on internal, and sometimes unhealthy strategies to cope with this stressful time. Healthcare providers who work with young families, and parental educational materials could highlight this dilemma and provide encouragement for couples to discuss and plan how they can support one another. This may include creating networks for new fathers to come together informally and build peer support. This study highlighted the importance of exploring ways to support fathers during their transition to parenthood and to encourage or empower those who need help to seek the help they need, especially since fathers are less likely to seek help from the most trusted person in their life, their partner, during her pregnancy and after birth.
